# Loss of Elongator- and KEOPS-Dependent tRNA Modifications Leads to Severe Growth Phenotypes and Protein Aggregation in Yeast

**DOI:** 10.3390/biom10020322

**Published:** 2020-02-18

**Authors:** Leticia Pollo-Oliveira, Roland Klassen, Nick Davis, Akif Ciftci, Jo Marie Bacusmo, Maria Martinelli, Michael S. DeMott, Thomas J. Begley, Peter C. Dedon, Raffael Schaffrath, Valérie de Crécy-Lagard

**Affiliations:** 1 Department of Microbiology and Cell Science, University of Florida, Gainesville, FL 32603, USA; lpollo@ufl.edu (L.P.-O.); bacusmo@ufl.edu (J.M.B.); manderson4@ufl.edu (M.M.); 2 Institut für Biologie, Fachgebiet Mikrobiologie, Universität Kassel, 34132 Kassel, Germany; roland.klassen@uni-kassel.de (R.K.); akif.ciftci@biochemie.uni-freiburg.de (A.C.); schaffrath@uni-kassel.de (R.S.); 3 Department of Biological Engineering, Massachusetts Institute of Technology, Cambridge, MA 02139, USA; kenezra@mit.edu (N.D.); msdemott@mit.edu (M.S.D.); pcdedon@mit.edu (P.C.D.); 4 The RNA Institute, College of Arts and Science, University at Albany, SUNY, Albany, NY 12222, USA; tbegley@albany.edu; 5 University of Florida Genetics Institute, Gainesville, FL 32608, USA

**Keywords:** tRNA modification, protein aggregation

## Abstract

Modifications found in the Anticodon Stem Loop (ASL) of tRNAs play important roles in regulating translational speed and accuracy. Threonylcarbamoyl adenosine (t^6^A37) and 5-methoxycarbonyl methyl-2-thiouridine (mcm^5^s^2^U34) are critical ASL modifications that have been linked to several human diseases. The model yeast *Saccharomyces cerevisiae* is viable despite the absence of both modifications, growth is however greatly impaired. The major observed consequence is a subsequent increase in protein aggregates and aberrant morphology. Proteomic analysis of the t^6^A-deficient strain (*sua5* mutant) revealed a global mistranslation leading to protein aggregation without regard to physicochemical properties or t^6^A-dependent or biased codon usage in parent genes. However, loss of sua5 led to increased expression of soluble proteins for mitochondrial function, protein quality processing/trafficking, oxidative stress response, and energy homeostasis. These results point to a global function for t^6^A in protein homeostasis very similar to mcm^5^/s^2^U modifications.

## 1. Introduction

Modifications of the four canonical bases found in the Anticodon Stem Loop (ASL) of tRNAs are critical for optimal decoding of mRNAs [1,2]. ASL modifications influence both decoding efficiency [3,4,5] and accuracy [6,7,8]. The roles of ASL modifications in decoding are complex and vary with the type of modification, its position in the ASL, the specific codon, and the organism [2]. To add to this complexity, the effect of a given modification is influenced by the codon context [9] and by the presence/absence of other modifications [10,11]. Deficiencies in the synthesis of many ASL modifications have been linked to disease of protein homeostasis in humans leading to a wide range of pathologies such as familial dysautonomia, nonsyndromic X-linked intellectual disability or microcephaly [12].

**Figure 1:**
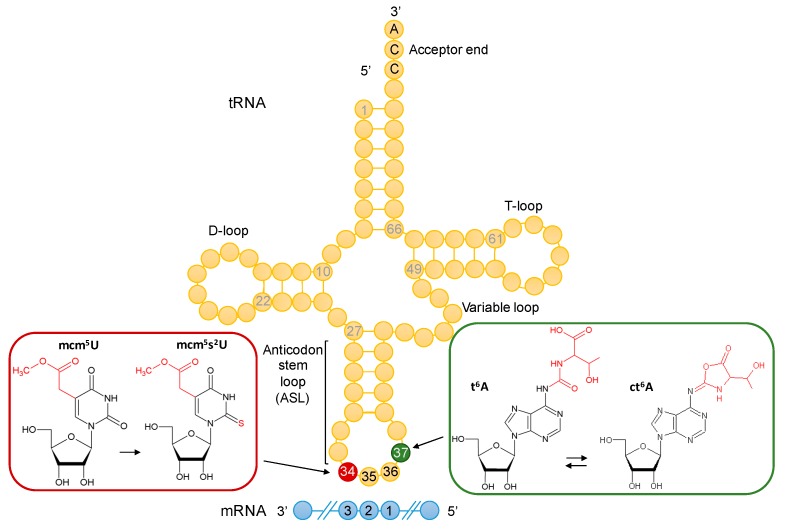
Anticodon-stem-loop (ASL) modifications in yeast tRNA. At the wobble position 34 (in red), 5-methoxycarbonyl-methyluridine (mcm^5^U) modifies tRNA^Arg^_UCU_, tRNA^Gln^_UUG_, tRNA^Glu^_UUC_ and tRNA^Lys^_UUU_, where it is further thiolated to 5-methoxycarbonyl methyl-2-thiouridine (mcm^5^s^2^U). Adjacent to the anticodon, at position 37 (in green), *N*^6^-threonyl-carbamoyl adenosine (t^6^A) modifies tRNAs that decode ANN codons (positions 1, 2, 3 of the mRNA codon) and is further modified to ct^6^A in several tRNAs. The tRNA molecule in yellow shows anticodon positions 34, 35, and 36. The mRNA molecule in blue highlights codon positions 1, 2, and 3. The structures of the modified bases were obtained from the Modomics database [31].

In eukaryotes, two modifications that greatly affect the structure of the ASL are *N*^6^-threonyl-carbamoyl adenosine (t^6^A37) and 5-methoxycarbonyl methyl-2-thiouridine (mcm^5^s^2^U34) [1] (Figure 1). Early structural studies showed that t^6^A is crucial for the prevention of U33-A37 pairing, thus stabilizing the anticodon open-loop configuration, and that both modifications are critical for correct pre-structuring of the ASL [13,14,15,16,17]. Deficiencies in both these modifications lead to severe neurological diseases [18,19,20,21,22], and the yeast *Saccharomyces cerevisiae* has been a long-standing model to study their synthesis and function [23,24,25,26,27,28]. In yeast, t^6^A is found at position 37 of tRNAs that decode ANN codons and is further modified to ct^6^A in several tRNAs such as tRNA^Lys^_UUU_ [29,30]. The mcm^5^U34 modification is found at the wobble position 34 of tRNA^Arg^_UCU_, tRNA^Gln^_UUG_, and in tRNA^Glu^_UUC_ and tRNA^Lys^_UUU_, where it is further thiolated to 5-methoxycarbonyl methyl-2-thiouridine (mcm^5^s^2^U). In yeast, only two tRNAs harbor both t^6^A and mcm^5^U34 modifications: tRNA^Lys^_UUU_ and tRNA^Arg^_UCU_. (Figure 1).

These complex modifications are synthesized in multi-step pathways [32] with key enzymes in multi-subunit complexes: the KEOPS complex for t^6^A synthesis [33] and the Elongator complex (Elp1–Elp6) for mcm^5^U [28,34]. While the mechanistic aspects of their synthesis are still being elucidated, most genes involved have been identified [32] and the pathways have been reconstituted fully [23] or partially [35,36] in vitro. This has allowed researchers to dissect the role of these modifications *in vivo*, where it has become apparent that defects in mcm^5^s^2^U34 and t^6^A37 synthesis in yeast gives rise to very similar phenotypes, including activation of the Gcn4 General Amino Acid Control (GAAC) response independently of Gcn2 and sensitivity to similar stresses [3,29,37]. The activation of the Gcn4 response seems to be a general response to tRNA modification deficiency in yeast as it was also observed in the absence of seven other modifications [9], but some of these cases have been shown to be in part Gcn2 dependent [38]. t^6^A37 and mcm^5^s^2^U34 are not positive determinants for their respective synthesis machineries, as the absence of one modification does not affect the presence of the other [39]. The t^6^A phenotypes are not suppressed by overexpression of any individual tRNA [39], whereas overexpressing tRNA^Gln^_UUG_ and tRNA^Lys^_UUU_ is sufficient to suppress most phenotypes caused by the absence of mcm^5^s^2^U34 [40]. In terms of the fine-tuning of translation speed, as measured by ribosome profiling, the absence of mcm^5^s^2^U34 in tRNAs leads to a clear reduction of translation speed at the cognate codons [4] consistent with its role in cognate codon binding that prevents tRNA rejection during ribosomal proofreading [41,42]. However, the response to t^6^A deficiency appears to be more nuanced, as translation speed increases or decreases depending on the specific codon [39]. While the absence of both modifications affects +1 frameshifting [24,41], they demonstrate different effects on misreading [36,43]. For example, the presence of t^6^A reduces misreading at UAG and UAA termination codons but increased misreading at error-prone codons. These effects were mostly independent of the presence of the mcm^5^ modification. Both mcm^5^ and s^2^ modification defects increase the misreading of the GGA Gly codon by tRNA^Glu^_UUC_ but have weaker effects on other mismatches.

A central theme emerging from studies of ASL modifications deficiencies involves a general disruption of proteome integrity and an increase in protein aggregation [2,44]. For instance, it is known that perturbations of translation speed can lead to protein misfolding directly by affecting co-translational folding [4,45], or indirectly through misincorporation of erroneous amino acids [45]. Accordingly, the absence of mcm^5^U and mcm^5^s^2^U in yeast and *C. elegans*, as well as the absence of t^6^A in yeast, has been shown to induce the formation of cellular protein aggregates [4,39,44]. In the case of mcm^5^s^2^U, strongly enhanced aggregate formation has been observed in the context of mutations that affect both mcm^5^U and s^2^U synthesis [4]. Aggregation phenotypes have also been observed in the absence of other tRNA modifications, such as queuosine (Q34) or m^2^_2_G26 in mammals [5,46,47]. The Unfolded Protein Response (UPR) is activated by t^6^A deficiency in higher eukaryotes [48] or mcm^5^U deficiency in different eukaryotic models but only mcm^5^U deficiency activates the UPR in *S. cerevisiae* [44,49]. Transcriptome analysis of yeast deficient in t^6^A reveals no UPR response [39], which is actually reduced in the absence of mcm^5^s^2^U in this strain [49]. Despite a growing body of research, few studies have systematically analyzed how the absence of tRNA modification affects the aggregation of yeast prions [50,51]. One would expect that the translation of the stretches of identical amino acids found in these specific proteins [52] is particularly sensitive to reduced translation speed. Indeed, synthesis of the Gln-rich prion Rnq1 is severely impaired by the absence of mcm^5^s^2^U34 and this defect can be rescued by overexpression of tRNA^Gln^_UUG_ [29], but it is not known if the absence of t^6^A affects the synthesis of the Asn/Thr rich prion SWI1 [53].

Several examples suggest a collaboration of different anticodon loop modifications in the maintenance of tRNA function [10,11,16,29,54]. Regarding the (c)t^6^A37 and mcm^5^(s^2^)U interactions, synthetic effects of partial loss of mcm^5^s^2^U or the cyclic form of t^6^A (ct^6^A) on yeast cell growth have been observed [29]. If the prevention of t^6^A cyclization in *tcd1* mutants did not result in strong aggregate induction, a combination of such defects with mcm^5^U or s^2^U deficiency did [29]. However, the combination of U34 hypomodification with the absence of t^6^A37 modification has never been studied, in part due to severe growth defects caused already by the loss of t^6^A alone [39]. In this work, we report that an *S. cerevisiae* strain lacking both t^6^A and mcm^5^/s^2^U34 modifications is greatly affected in growth and morphology, with an observed synthetic lethality in specific conditions, as well as additive effects in protein aggregation and +1 frameshifting phenotypes. As the proteomic analysis of t^6^A deficiency has previously only been performed in bacteria [55], we also compared soluble and insoluble (or “aggregated”) fractions of the yeast proteome between WT and t^6^A-deficient strains. Our proteomic results describe the consequences of perturbing translation through ASL modification deficiency and provide insights into correlating shifts in codon usage.

## 2. Materials and Methods

### 2.1. Strains, Plasmids and Growth Assays

The strains used and generated in this study are listed in Table 1. Gene replacements were verified with forward/reverse primers positioned outside of the target loci (Supplemental Table S1). Cultivation of the different strains with yeast nitrogen base (YNB)/yeast peptone dextrose (YPD) as well as yeast transformations were performed using standard methods [56]. A BY4741 *elp3::SpHIS5* mutant was generated by marker swap using BY4741 *elp3::KANMX4* and pUG27 [57]. GFP tagging of *HSP104* was done using pFA6a-GFP-*natMX* [58]. Crosses were done by patching haploid BY4742 (*MATα lys2*) derived and BY4741 (*MATa met15*) strains on –Met –Lys media. Heterozygous diploids were first subcloned on selective minimal media, then on YPD and finally put on sporulation media (20 g/L potassium acetate, 1 g/L glucose, 2.5 g/L yeast extract 20 g/L agar plus required supplements to cover auxotrophic markers). Sporulation was monitored microscopically. Cells were recovered from sporulation plates and resuspended in 200 µL sterile water to which 5 to 20 µL zymolyase stock solution (zymolyase 20T, 5 mg/mL) was added. Following incubation for 5 to 10 min at 37 °C, 1 mL of ice-cold sterile water was added, and 20 µL of digested cells placed on the edge of a YPD plate. Tetrads were dissected using a Singer MSM400 micromanipulator (Singer instruments, Roadwater, Watchet, UK) and genotypes of individual spores assessed by diagnostic PCR analysis and checking auxotrophic markers and G418 resistance on appropriate media.

### 2.2. Plasmid Construction

pYX142-mtGFP [60] was used as a backbone for the construction of pJMB21. To monitor expression on both the N- and C-terminal ends, a HA-tag flanked by two new multiple cloning sites (MCS) were introduced into pYX142-mtGFP (Figure S1). This new construct allowed for the expression of proteins containing an N-terminal HA-tag with a C-terminal GFP fusion. Gene synthesis and plasmid construction were sourced through GenScript (Order 702065-3). The N-terminal end of SWI1 (residues 1-556) was synthesized (GenScript) and inserted into pJMB21 between the SbfI and AscI restriction sites to give plasmid pJMB21::SWI1.

### 2.3. Detection of HA-SWI1-GFP Fusion in t^6^A Deficient Strains

Competent wild-type (BY4741) and two t^6^A deficient strains (*gon7* and *sua5* mutants) were transformed with pJMB21 and pJMB21::SWI1 using the Frozen-EZ Yeast Transformation II Kit (Zymo Research, Cat#T2001, Irvine, CA, USA) and selected in minimal synthetic defined base (SD) with dropout supplements (-leucine) (SD-Leu) (Takara, Cat# 630,411 and 630414, Mountain View, CA, USA). The transformants were grown overnight and sub-cultured in SD-Leu to reach the early exponential phase (OD_600_ of 0.6). The cell pellets were stored at −80 °C until protein extracts were prepared. The cell pellets were resuspended in water and normalized based on the OD600 (equivalent to 5 mL culture at OD_600_ 0.6). To facilitate the permeabilization of yeast cells, the pellets were washed in 2 M LiOAc and then in 0.4 M NaOH (kept 5 min on ice with each solution) [61]. Extracts were prepared by boiling the cells for 10 min in 200 µL of SDS loading buffer (10% SDS, 250 mM Tris-Cl pH 6.8, 500 mM DTT, 25% glycerol, bromophenol blue) and then loaded (12 µL/well) in 12% acrylamide gel. For Western blot, proteins were transferred to a PVDF membrane and probed with 1:1000 HA Epitope Tag Antibody, HRP conjugate (Thermo Fisher Scientific, Cat# 26183-HRP, Rockford, IL, USA).

### 2.4. Detection of Protein Aggregates by Different Methods

Protein aggregates were first isolated as described previously [29]. Cell pellets were obtained from 50-mL YPD cultures of BY4742 derivatives grown to OD_600_ = 1.0. Cells were broken by sonication and 4 mg total protein was subjected to centrifugation and washing [29]. The remaining aggregate pellet was subsequently boiled in SDS sample buffer and separated on NuPAGE Bis-Tris 4–12% gradient gels. For comparison, 25 µg total protein was separated on identical gels.

For visualization of aggregates within cells, strains expressing a GFP tagged variant of the aggregate binding protein Hsp104 were generated by genomic tagging of HSP104 and subsequent crossing to *sua5 elp3* followed by tetrad dissection. WT and sua5/elp3 single and double mutant strains carrying the genomic *HSP104-GFP* fusion were grown to log phase, washed twice in sterile water and visualized in phase contrast and fluorescence optics using an Olympus BX53 microscope (Olympus, Hamburg, Germany).

The final method to quantify protein aggregation used [^35^S]-labeling. BY4742 cells (WT, *sua5* mutant, and *sua5 elp3* double mutant) were grown from a single colony in 10 mL of complete medium lacking Met and Cys with 5 µL of 100 mM Met and Cys added (50 µM final). Overnight cultures were diluted to 0.1 OD_600_ in 5 mL of complete medium lacking Met and Cys with the addition of 2.5 µL of 100 mM Met and Cys (50 µM final) and 3 µL (33 µCi) of [^35^S]-Met and of [^35^S]-Cys. The three strains grew at different rates, so cultures were grown to a constant optical density (1.2 OD_600_), which required 7 h for WT, 26 h for *sua5*, and 68 h for *sua5 elp3*. Three normalized aliquots for each strain (equivalent to OD_600_ = 1.5 per mL) were prepared as technical replicates. Cells were pelleted by centrifugation at 7000× *g* for 10 min at 4 °C and 600 µL of the supernatant was removed; the remainder of the supernatant was discarded. One-half of the supernatant (300 µL) was placed in a 3000 Dalton spin filter and centrifuged for 10 min at 16,100× *g* at 4 °C. The 300 µL of remaining input and the flow-through were saved for scintillation counting. To the retentate in the spin filter, 300 µL of PBS was added and the sample centrifuged for 10 min at 16,100× g, with the flow-through discarded. This was repeated one time. The empty 3000 Dalton spin filter membrane was saved for scintillation counting. To the cell pellet remaining from above 600 µL of PBS was added and the pellet was resuspended. Following centrifugation at 7000× *g* for 10 min at 4 °C, the supernatant was discarded, the pellet washed again with 600 µL of PBS, discarding the supernatant. The cell pellet was resuspended in 700 µL of PBS and 5 µL of lyticase (50 units) was added with mixing and incubation at RT for 40 min. The samples were then transferred to MP Bio Lysing Matrix C (1.0 mm) tubes and caps were tightened tightly. Samples were processed on a Thermo FastPrep (FP120) bead-beater 3 times at speed 6.0 for 30 s with a 30 s pause between runs. Samples were then centrifuged at 200× *g* for 10 min at 4 °C to remove glass beads and the supernatant was transferred to 2 mL plastic tubes, to which 300 µL of PBS was added and the samples vortexed. Following centrifugation at 200× *g* for 10 min at RT, the supernatant was transferred to 2 mL plastic tubes and the glass beads were saved for scintillation counting. The supernatants were now centrifuged at 16100× *g* for 10 min at RT and the new supernatants transferred to new 2 mL plastic tubes and the pellets in original tubes saved for scintillation counting. The new supernatants were again centrifuged at 16,100× *g* at RT to completely clear the samples before transferring supernatant to a 3000 Dalton spin filter followed by centrifugation at 16,100× *g* for 10 min at RT. The remaining supernatant and the flow-through from the 3000 Dalton spin filter were saved for scintillation counting. All samples were subjected to scintillation counting in an LS 6500 Beckman Coulter Scintillation counter. Scintillation counting data were normalized to total protein levels measured by the BCA Assay (Bio-Rad, Hercules, CA, USA).

### 2.5. Proteomic Analyses

#### 2.5.1. Isolation of Soluble and Insoluble Proteins

Soluble and insoluble protein fractions were isolated from yeast cells as described by Koplin et al., 2010, with a few modifications [62]. Briefly, logarithmically growing cells cultivated in MM-His (50 OD_600_ units) were harvested at 200× *g* for 10 min at 4 °C and the resulting cell pellets were washed with ice-cold phosphate-buffered saline (PBS) and frozen at −80 °C. To prepare cell lysates, pellets were resuspended in 500 µL of lysis buffer (20 mM Na-phosphate, pH 6.8, 10 mM DTT, 1 mM EDTA, 0.1% *v/v* Tween, 1 mM PMSF, Roche protease inhibitor cocktail, 3 mg/mL lyticase and 1.25 U/mL benzonase), and incubated at 30 °C with mild shaking for 30 min. Glass beads were used to disrupt cells using a Precellys 24 disrupter; 2 cycles of 25 s at 6500 rpm; samples were kept on ice between each cycle. Cell lysates were then centrifuged for 20 min at 200× *g* at 4 °C and supernatant fractions were aspirated, analyzed by the BCA method [63] and adjusted to equimolar protein concentrations (4.8 mg/mL for protein gels and 4 mg/mL for LC-MS/MS analysis) across samples. Membrane and aggregated proteins were isolated from this supernatant fraction by centrifugation at 16,000× *g* for 20 min at 4 °C. Following this round of centrifugation, resulting supernatant fractions were aspirated and membrane proteins were removed by resuspending aggregated proteins in 2% NP-40 (in 20 mM Na-phosphate, pH 6.8, 1 mM PMSF and Roche protease inhibitor cocktail), disrupting the mixture by probe sonication (6-times for 5 s at cycle 0.1 and amplitude 20%), and centrifuging the mixture at 16,000× *g* for 20 min at 4 °C. This process was repeated twice, after which final insoluble protein fractions were washed with buffer lacking NP-40 (probe sonication, 4-times for 5 s at cycle 0.1 and amplitude 20%). For electrophoretic analyses, the pellets and samples of soluble proteins were boiled in 1X Laemli sample buffer, separated by SDS-PAGE (14%), and resolved by Coomassie staining. For LC-MS proteomics analyses, samples of soluble proteins and insoluble protein aggregates were precipitated with TCA (100% *w*/*v*) by adding 1 volume of TCA to 4 volumes of protein solution followed by incubation at 4 °C for 30 min. Precipitated protein samples were washed with 200 µL of ice-cold acetone (9:1 *v*/*v*), and pellets were allowed to air dry at RT. Dried protein pellets were resuspended in 1 mL of 10 mM TEAB and quantified by BCA Assay (Bio-Rad) before further processing for MS/MS analysis.

#### 2.5.2. Protein Processing, Labeling with Isobaric Tags, and Peptide Fractionation

Protein samples were aliquoted (100 µg per sample), dried by vacuum centrifugation, reconstituted in 100 mM TEAB and 10% acetonitrile (*v*/*v*) by bath sonication, and digested with trypsin in a 1:30 (*w*/*w*) ratio overnight at 37 °C. Aliquots of resulting protein digests (from 100 µg of total protein) were then labeled with TMT 6-plex reagents according to the manufacturer’s protocol. Labeled peptides (5-µL aliquots) from each biological replicate were combined to reconstitute a full 6-plex label set and subjected to preliminary qualitative analysis on a Thermo Scientific EASY-nLC 1200 interfaced to a Thermo Scientific Q Exactive Hybrid Quadrupole-Orbitrap mass spectrometer (Thermo Fisher Scientific, Waltham, MA, USA) Median total ion intensities for each label were calculated and used to normalize volumetric mixing of respective labels, so as to avoid signal suppression or bias from any one label. After combining labels into 6-plex sets, samples were desalted with C18 SpinTips (Protea), dried by vacuum centrifugation, and reconstituted in IPD buffer (Agilent) without glycerol. Isoelectric focusing was performed from pH 3 to 10 over 24 wells on an Agilent 3100 OFFGEL fractionator according to the manufacturer’s protocol (OG24PE00). Each of the 24 fractions was collected, dried by vacuum centrifuge, resuspended in 0.1% formic acid in water, and analyzed by nano-LC-MS/MS.

#### 2.5.3. LC-MS Analysis of the Aggregated Yeast Proteome

TMT proteomics experiments were performed on an Agilent 1200 nano-LC-Chip/MS interfaced to an Agilent 6550 iFunnel Q-TOF LC/MS. The LC system consisted of a capillary pump for sample loading, a nanoflow pump, and a thermostated microwell-plate autosampler. The HPLC-Chip configuration consisted of a 160-nL enrichment column and a 150 mm × 75 µm analytical column (G4340-62001 Zorbax 300SB-C18). The following mass-spectrometry grade mobile phases (Burdick & Jackson) were used: 0.1% formic acid in water (solvent A), and 0.1% formic acid in acetonitrile (solvent B). A 130-min linear gradient was used for HPLC separation with 10 min for column washing and equilibration between runs. Samples (1–2 µL injections) were loaded onto the enrichment column at 3% (*v*/*v*) B at flow rates of 3 µL min-1. The analytical gradient of solvent B was performed at a constant flow rate of 0.3 µL min-1 using the following solvent transitions on the nanoflow pump: 0–1 min, held at 1% (*v*/*v*); 1–10 min, 1–15%; 10–101 min, 15–35%; 101–121 min, 35–75%; 121–123 min, 75–98%; 123–126 min, held at 98%; 126–127 min, 98–1%; 127–130 min, held at 1%. The Q-TOF was operated at high sensitivity (4 GHz) in positive ion mode with the following source conditions: gas temperature 350 °C, drying gas 13 L min-1, fragmentor 360 V. Capillary voltage was manually adjusted between 1800 to 2150 V to maintain a steady nanospray plume. Data were acquired from 300 to 1700 *m*/*z* with an acquisition rate of 6 spectra s^−1^ in MS mode, and from 50 to 1,700 *m*/*z* with an acquisition rate of 3 spectra s^−1^ in MS/MS mode. A peptide isotope model (charge state 2+) was used to detect a maximum of 20 precursors per cycle at a minimum threshold of 25,000 counts/spectra at a narrow isolation window (~1.3 *m*/*z*). Sloped collision energy (C.E.) was used to maximize collision-induced dissociation of detected isobarically tagged peptides according to the following rules: charge state 2+ C.E. slope 4.2, offset 3.5; charge states ≥ 3+ C.E. slope 4.2, offset 4.

LC-MS data were extracted and evaluated for quality using the MFE algorithm in MassHunter Qualitative Analysis software (v B06.00). Test injections (3–4) from each fraction of the first technical replicate were used to optimize injection volumes for second and third biological replicates with the aim of maximizing the number of extracted molecules with peptide-like features. For each fraction, the MFE list of molecular ions was exported and used to exclude the spectral acquisition of these ions in subsequent technical replicates. Each of the 24 fractions from biological triplicates was injected in technical duplicate—spectra generated from technical replicate #1 were acquired without the use of an exclusion list, whereas spectra generated from technical replicates #2 and #3 were acquired with the exclusion list. Data from MassHunter Qualitative Analysis was exported to Mass Profiler Professional (v B03.00) for analysis of technical reproducibility. This process was repeated for all three biological replicates. Mass spectra were processed using Spectrum Mill (Agilent, v B06.00) and Scaffold Q+ (v Scaffold_4.8.8), and quantified protein associations were manually analyzed by binning and averaging peptide quantities across related protein groups in Excel. For the insoluble fraction, this analysis produced 327,549 spectra that were assigned to 6421 identified *S. cerevisiae* proteins (peptide threshold: < 1% FDR) and 5109 proteins with quantifiable peptides across all three biological replicates (protein threshold: > 99% confidence, min 2 peptides). The soluble fraction yielded 42,611 spectra assigned to 4890 identified *S. cerevisiae* proteins, of which 3814 were quantifiable across all three biological replicates. Proteomics data are available at Chorus Project (https://chorusproject.org/pages/index.html).

#### 2.5.4. Proteomics Data Analysis

Proteomics data were used to calculate fold-change values (*sua5*/WT) for the sets of soluble and insoluble proteins. Differentially expressed proteins in WT and *sua5* were then analyzed for various physicochemical properties using either Saccharomyces Genome Database data (https://www.yeastgenome.org) or publicly available datasets: isoelectric point [64], codon adaptation index [65], protein half-life [66,67], protein abundance [66,68], molecular weight [69], and hydrophobicity [69]. Total proteins in each fraction and differentially expressed proteins were analyzed for Gene Ontology category enrichment [69].

#### 2.5.5. Codon Usage Analysis

Gene-specific codon usage data detailing the number of times each codon was used in a gene and the frequency of each codon, relative to other codons for the same amino acid in said gene, was previously described [70]. Codon frequency trends for sets of transcripts were determined by taking the average frequency value of each codon in the group. The number of times a specific dicodon was found in each gene was determined using slight modifications to the original GSCU algorithm developed for gene-specific (mono) codon usage analysis [71]. Briefly, each gene was analyzed from start to stop codon for the instance of a specific two codon combination, data were tabulated for each gene, and then the next gene was analyzed. The resulting dicodon data were then sorted into groups genes with identical instances of specific dicodon combinations and graphed in Excel. Gene ontology analysis was performed using utilities found in STRING (https://string-db.org/) [71].

#### 2.5.6. Statistical Analyses

Differentially expressed proteins were determined using one-way ANOVA using Bonferroni multiple testing correction. Differential abundance of proteins was analyzed by a random-effects Bayes model using the BETR algorithm in MeV (http://www.tm4.org/mev.html). Interpretations of the relationships between codon usage predictors (codon frequency) and protein up-or down-regulation (log_2_ mean fold-change) were analyzed using a partial least squares regression (PLSR) algorithm in The UnscramblerX (v10.4, CAMO Software). Outlier loadings that could cause over-fitting were removed by inspection of variable residuals and leverages. Validation was performed using cross-validation and the significance of variables determined by Marten’s uncertainty test. The Root Mean Square Error of Prediction (RMSEP), slope, and correlation coefficient of predicted versus measured correlation line were used to evaluate the efficiency of the applied regression model.

### 2.6. Whole-Cell Analyses

For microscopic detection of nuclei, cells were first fixed in 70% ethanol for 10 min and subsequently resuspended in 1 µg/mL 4′,6-diamidino-2-phenylindole (DAPI). After incubation for 1 h in the dark, cells were washed with sterile water and observed in phase contrast and fluorescence optics using an Olympus BX53 microscope. +1 frameshift assays utilized constructs with the frameshift site CUU-AAA-C [72] and were carried out as described previously [49].

## 3. Results

### 3.1. Absence of t^6^A and mcm^5^U Leads to Additive and Possibly Synergistic Translation Defects

**Figure 2:**
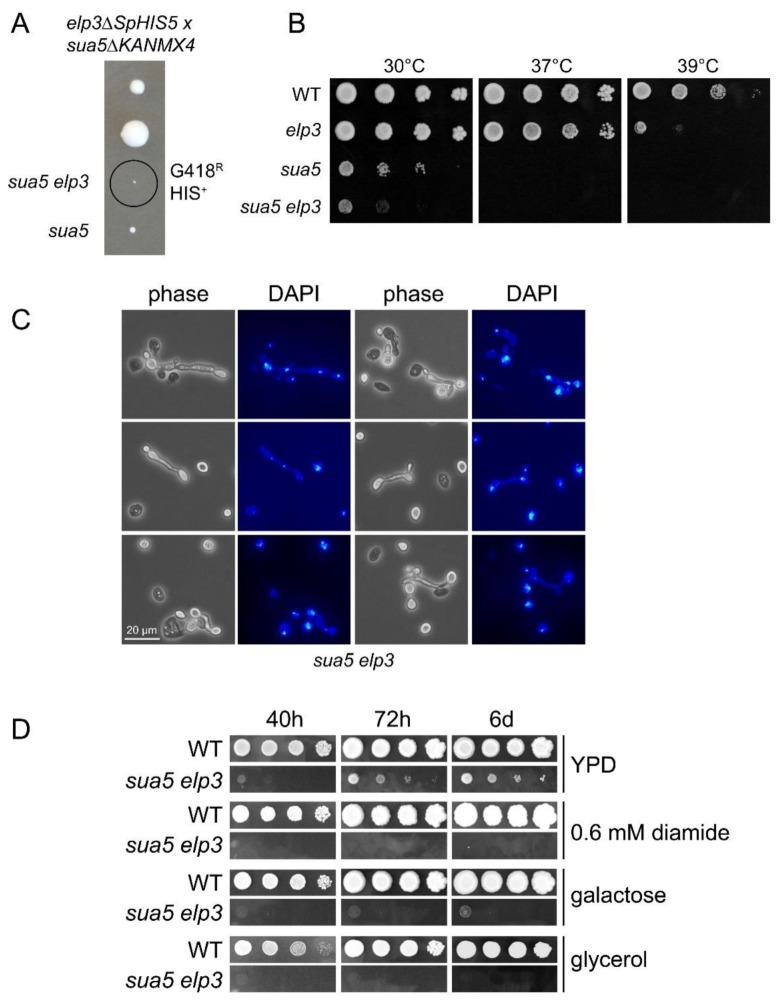
Synthetic phenotypes in the genetic background of *sua5 elp3* mutants. (**A**) Tetrad analysis of a *sua5::KANMX4/SUA5 elp3::SpHIS5/ELP3* diploid strain, generated by crossing BY4741 *elp3::SpHIS5* and BY4742 *sua5::KANMX4*. The genotype of indicated spores was determined by phenotypic analysis (-HIS media, G418 media) and diagnostic PCR. G418R: Geneticin resistance; HIS+: Histidine prototrophy. (**B**) Serial dilution spot assay of indicated strains on YPD plates, which were incubated at 30 °C, 37 °C, or 39 °C for 48 h. (**C**) Elongated bud morphology and nuclear segregation defect of the *sua5 elp3* strain. Cells were ethanol fixed and stained with DAPI before phase-contrast and fluorescence microscopy. (**D**) Serial dilution spot assay of indicated strains on YPD, YPD containing 0.6 mM diamide, yeast peptone galactose or yeast peptone glycerol medium. Plates were photographed after the indicated incubation times.

**Figure 3:**
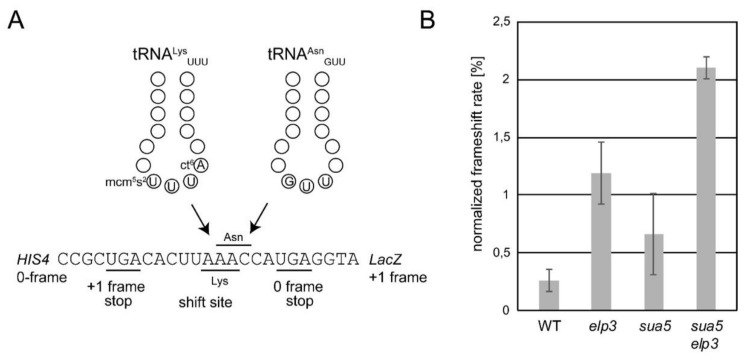
Programmed +1 frameshifts are triggered in the *sua5 elp3* mutant. (**A**) Schematic representation of the utilized +1 frameshift reporter constructs harboring a tRNA^Lys^_UUU_ dependent frameshift site [49,72]. In the event of diminished A-site binding activity of tRNA^Lys^_UUU_, tRNA^Asn^_GUU_ may instead read the Asn codon in the +1 shifted reading frame, ultimately allowing expression of the reporter LacZ which is in the +1 frame relative to the Lys AAA codon. (**B**) Measurement of +1 frameshift rates by employing the reporter described in (**A**) and a control construct, as detailed previously [49,72]. These assays were conducted on three independent cultures per strain and each culture measured using two technical replicates.

*S. cerevisiae* strains carrying a deletion in *ELP3*, which encodes the catalytic subunit of the Elongator complex, lack mcm^5^U in tRNA [28] while strains with deletions of *SUA5* encoding the first enzyme of the t^6^A synthesis pathway, threonylcarbamoyl-AMP synthase, lack t^6^A [25]. To test whether *elp3* and *sua5* mutations are in fact synthetically lethal, we sporulated a heterozygous *sua5 elp3* double mutant (Figure 2A). Spores containing both mutant alleles are able to germinate but form visible colonies only several days after the appearance of colonies from spores without the *sua5* allele alone. Serial dilution growth assays indeed revealed a further enhanced growth defect of the *sua5 elp3* strain as compared to the *sua5* single mutant (Figure 2B). Microscopic analysis of cell shape and nuclei distribution revealed morphological defects and mis-segregated nuclei in the *sua5 elp3* (Figure 2C). In addition, the obtained *sua5 elp3* strain is unable to grow in the presence of various exogenous stressors such as elevated temperature, diamide, or alternative carbon sources (Figure 2B,D). As the presence of mcm^5^s^2^U in yeast tRNA^Lys^_UUU_ suppressed +1 frameshift events, and t^6^A is also involved in reading frame maintenance [24,72], we analyzed a potential cumulative effect on +1 frameshift levels in the generated *sua5 elp3* mutants. We utilized the previously described frameshift sequence in which a +1 ribosomal shift can be induced by weakened A-site binding of tRNA^Lys^_UUU_ [72]. In both *sua5* and *elp3* single mutants, elevated +1 shift levels were observed, which further increase in the *sua5 elp3* double mutant (Figure 3). Thus, mcm^5^s^2^U and t^6^A indeed independently contribute to reading frame maintenance.

### 3.2. The Absence of both ASL Modifications Drastically Increases Formation of Protein Aggregates

**Figure 4:**
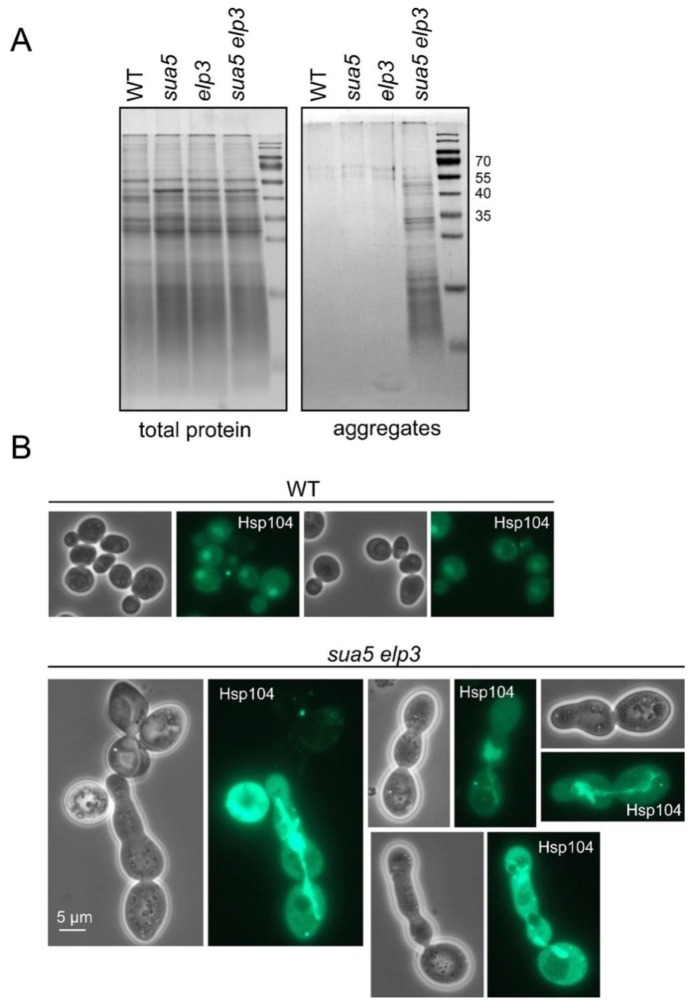
Aggregate formation in the *sua5 elp3* double mutant. (**A**) Protein extracts of indicated strains were generated and analyzed by SDS PAGE before (total protein) and after enrichment of aggregates, as described previously [4,29]. Aggregates were isolated twice from each strain. Note that the method used for the solubilization of aggregated proteins used here is not as harsh as that used for the [^35^S] labeling and proteomics analyses (Figure S3). (**B**) Expression Hsp104-GFP from its natural genomic locus in WT and *sua5 elp3* backgrounds. The *HSP104-GFP* allele was introduced into *sua5 elp3* by crossing and tetrad dissection.

Hence, we speculated that the absence of t^6^A and mcm^5^/s^2^U in the generated *sua5 elp3* strain would result in a further increase of protein aggregation if aggregates result from ribosomal pausing and pausing results from tRNA A-site binding deficiency. As the +1 frameshift measurements are consistent with cumulative A-site binding defects of tRNA^Lys^_UUU_ in *sua5 elp3*, a cumulative effect on protein aggregation could be expected if the latter indeed occurs because of the A-site binding defect. To test this, we utilized a previously established aggregate enrichment protocol [62] and compared amounts of aggregated proteins in wild type, *sua5*, and *elp3* single mutants with the double mutant. As shown in Figure 4A, there is indeed a strongly increased amount of protein aggregates in the double mutant.

Cellular protein aggregates were previously visualized using GFP tagged Hsp104, an aggregate binding chaperone [73,74]. To address Hsp104 localization in the *sua5 elp3* strain which accumulates protein aggregates, we introduced an *HSP104-GFP* allele into this background and compared GFP signals between wild type and the mutant. The wild-type shows a GFP signal typical of the known nucleo-cytoplasmic distribution and only rarely shows small bright foci indicative of cytoplasmic protein aggregation [75] (Figure 4B). In the *sua5 elp3* mutant, however, bright signal accumulations were observed particularly in morphologically aberrant cells, with aggregates often being extended along the axis of polarized growth and encompassing two or more of the non-separated individual cells (Figure 4B). This may indicate that aggregate formation and extension from the mother into the daughter cell may contribute to the repeated failures in cytokinesis. Similar Hsp104 signal accumulations and morphologically aberrant cells were occasionally detectable for the *sua5* single mutant as well but not for an *elp3* single mutant (Figure S2).

To more accurately assess tRNA modification-dependent protein aggregation and translation in general, we used [^35^S]-labeled methionine and cysteine to quantify protein expression in WT, *sua5*, and *sua5 elp3* strains. The significantly different growth rates of the strains were accommodated by growing cultures to the same optical density and then isolating and quantifying [^35^S] in soluble and insoluble proteins in the strains. As shown in Supplementary Figure S3A, the three yeast strains showed progressively diminishing [^35^S] in soluble and insoluble proteins in all fractions in the order WT > *sua5* > *sua5 elp3*. However, total protein concentration in the soluble fractions was similar for the three strains (Supplementary Figure S3B). Coupled with similar [^35^S] labeling in all fractions, this suggests that incorporation of [^35^S]-labeled Met and Cys was reduced in the two mutant strains. This is supported by the observation of reduced [^35^S] labeling of proteins in SDS-PAGE gels run with samples normalized for either protein concentration or [^35^S] content: the amount of [^35^S] signal per unit protein is highest in WT and progressively reduced in the mutant strains (Supplementary Figure S3C). This unexpected dilution of the specific activity of [^35^S] obviates the use of [^35^S] labeling to quantify protein aggregation differences among the strains.

### 3.3. Loss of t^6^A Leads to Global Defects in Protein Folding and Mitochondrial Assembly

To obtain a more granular view of the effects of loss of t^6^A on translation, we performed a quantitative analysis of the soluble and aggregated (insoluble) proteomes of WT and *sua5* strains (the *elp3 sua5* mutant being too crippled for reproducible proteomic analysis). Protein aggregates were isolated by several rounds of sonication and differential centrifugation of cell lysates using the detergent buffer. Subsequent TMT-based quantitative proteomics resulted in the coverage of 60–80% of the yeast genome, with 3813 proteins quantified in the soluble fraction, 5108 proteins quantified in the insoluble fraction, and 1864 proteins in a whole-cell extract, with very similar broad distributions across all major gene ontology categories for the three datasets (Figure S4). Analysis of fold-change data (*sua5*/WT) for the sets of soluble and insoluble proteins revealed the following numbers of significantly altered proteins (+30% fold-change relative to WT values, *p* < 0.05): 93 increased and 43 decreased in the soluble fraction of the mutant, and 16 increased and 7 decreased in the insoluble fraction (Table S2). An unexpected result from this analysis was the small number of proteins significantly increased or decreased in the protein aggregates (i.e., insoluble) from the *sua5* mutant compared to WT cells. Given the evidence for “aggregation-prone” proteins and differential aggregation of proteins based on physicochemical properties [76], we undertook an analysis of the properties associated with the significantly up- and down-regulated proteins in the soluble and insoluble fractions from WT and *sua5* strains. As shown in Supplementary Figure S5, we found no significant differential associations of protein fractions for isoelectric point (pI), codon adaptation index (CAI), protein size (Da), protein half-life, hydrophobicity (GRAVY score), and abundance. Of note, the limited number of significantly differentially expressed proteins in the insoluble fraction constrains the statistical confidence of group-averaged physicochemical analyses of this group.

In light of the lack of physicochemical distinctions of the aggregated proteins in the *sua5* mutant and WT strains, we next analyzed the aggregated and soluble proteins for differences in function and codon usage patterns. As shown in Table 2, there were significant enrichments in several GO categories related to protein folding and stress response for up-regulated proteins in the soluble fraction. A more granular look at specific proteins enriched in GO categories is shown in Table 3. Here we see enrichment in mitochondrial assembly/function, protein quality processing/trafficking, oxidative stress response, and energy homeostasis.

**Figure 5:**
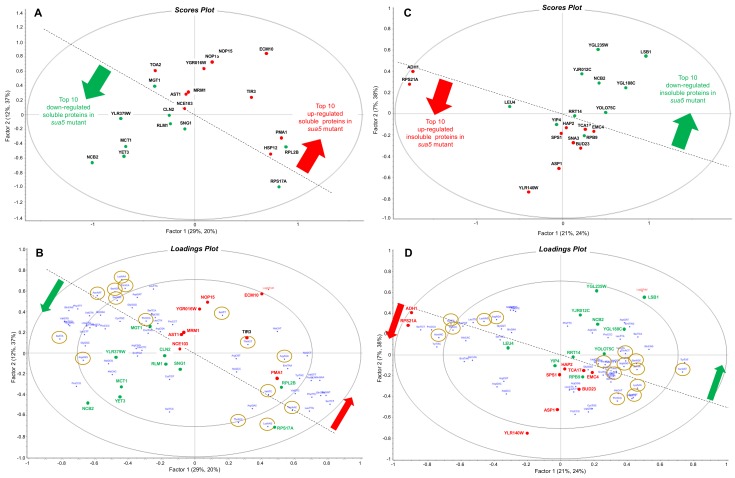
Partial least squares regression analysis of the association of codon usage with up- and down-regulated proteins in cells lacking Sua5. Soluble (**A**,**B**) and insoluble proteins (**C**,**D**) were isolated from wild-type and *sua5* strains of *S. cerevisiae* and subjected to quantitative proteomics analysis. Codon usage in the 10 most up- and down-regulated proteins in the *sua5* strain compared to wild-type was quantified using the codon utilization tool [82]. Partial least squares regression analysis was performed on the proteomic fold-change values and the codon usage data, with the resulting scores plots (**A**,**C**) and loadings plots (**B**,**D**) colored as follows: red, up-regulated proteins; green, down-regulated proteins; gold circles, ANN codons read by t^6^A-containing tRNAs; dotted line highlights the distinction between up- and down-regulated proteins. Some proteins from the scores plot (those that do no mask codons) are transposed to the loadings plot to highlight codon associations.

Given the association of t^6^A at position 37 of tRNAs that read ANN codons, we performed an analysis of the codon usage patterns in up- and down-regulated proteins in the soluble and insoluble fractions (*sua5*/WT). Figure 5A shows the scores plot in a principal components analysis of the 10 most up-regulated proteins (red) in the soluble fraction from the *sua5* mutant (relative to WT) and the 10 most down-regulated soluble proteins (green), with a clear segregation of the up- and down-regulated proteins highlighted by the dotted line. While there is no clear bias in the use of t^6^A-dependent ANN codons in the loadings plot (brown circles in Figure 5B), there are several pairs of synonymous codon partners that strongly distinguish the up- and down-regulated proteins (Figure 5B): ProCCG/down and ProCCA/up; SerTCC/down and SerAGC-AGT/up; HisCAC/down and HisCAT/up; AlaGCG/down and AlaGCT/up; ArgAGG/down and ArgAGA/up; IleATA/down and IleATT/ATC/up; GlyGGC/down and GlyGGT/up. We have observed this type of biased use of synonymous codon pairs in differentially regulated genes in yeast, bacteria, and human cells, with a strong link to coordinated changes in the tRNA pool and tRNA wobble modifications [77,78,79,80,81].

Parallel codon usage behavior was observed in the insoluble proteins in the *sua5* mutant, with no apparent bias in the use of t^6^A-dependent ANN codons in up- and down-regulated proteins. As shown in the scores plot in Figure 5C, the 10 most significant up- and down-regulated proteins in the insoluble fraction are distinguished from each other, though not as clearly as the soluble fraction. The loadings plot in Figure 5D shows the codon biases most strongly associated with the up- and down-regulated proteins: AlaGCG/down and AlaGCC/up; ArgCGC/down and ArgCGT/up; and AspGAT/down and AspGAC/up.

**Figure 6:**
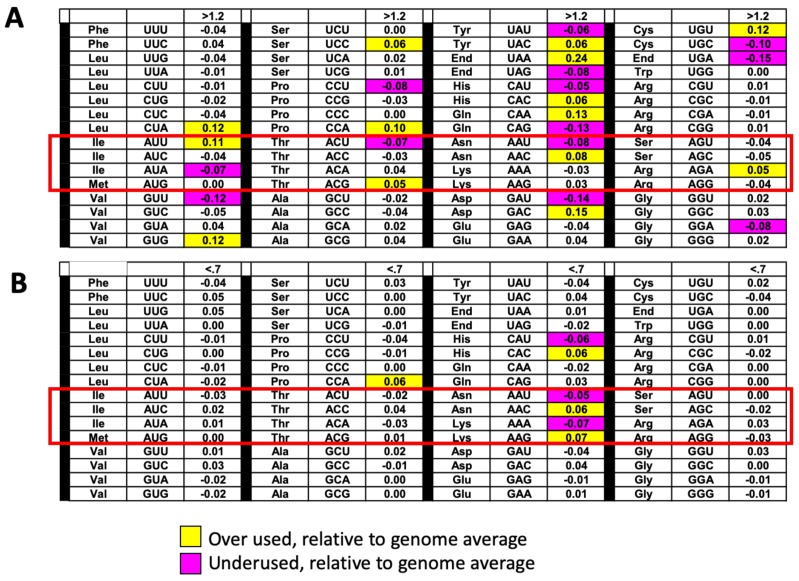
Codon usage trends in groups of transcripts corresponding to proteins regulated in *sua5* cells. (**A**) Insoluble proteins up-regulated (fold change > 1.2, *p* < 0.05) in *sua5* cells have corresponding transcripts that over-use AUU, ACG, AAC and AGA codons. (**B**) Soluble proteins down-regulated (fold change < 0.7, *p* < 0.05) in sua5 cells have corresponding transcripts that over-use AAC and AAG codons. The color-coded table describes the increased (yellow) or decreased (purple) codon frequency changes in a group of regulated proteins relative to genome averages (Codon^-Group^ - Codon^-Genome^) for each of 64 codons, with white boxes describing changes less than 0.05. The t^6^A dependent codons are the ones in the red-box.

We have analyzed the codon biases for open reading frames corresponding to the up-regulated proteins in the insoluble and down-regulated proteins in the soluble fraction, and while there were notable biases (Figure 6), clear linkages to ANN codons were not identified. We have observed that there are a distinct set of proteins whose open reading frames (ORFS) only contain ANN codons for arginine (AGA and AGG) and STRING analysis has identified gene ontology (GO) terms (ATP metabolic process, purine ribonucleoside monophosphate metabolic process, ribonucleoside monophosphate metabolic process, electron transport chain, purine ribonucleotide metabolic process, oxidative phosphorylation, ribonucleotide metabolic process, ribose phosphate metabolic process, mitochondrial ATP synthesis coupled electron transport) that detail mitochondrial based ATP production and ribonucleoside synthesis are significantly enriched (*p* < 10^–9^) (Table 4). We also analyzed all yeast ORFs to identify how many contain AGN-AGN dicodons (Figure S6A), with 2,995 containing 0, and the rest containing anywhere from 1 to 14 of these dicodons. Further analysis of AGN-AGN dicodons was assessed in ORFs only using ANN codons for arginine, with 211 of these containing 0 AGN-AGN dicodons and the remaining 57 containing 1 to 4 (Figure S6B). These results support the idea that most ORFs using AGN-AGN dicodons also use the other codons for arginine (CGN).

To summarize the proteomics results, the loss of t^6^A resulted in global defects in protein folding, with aggregated and soluble proteins sharing similar physicochemical properties and use of t^6^A-dependent codons (ANN) in parent transcripts. However, loss of t^6^A caused a stress response apparent as increased expression of mitochondrial assembly/function, protein quality processing/trafficking, oxidative stress response, and energy homeostasis proteins in the soluble fraction. This stress response was associated with non-ANN codon biases, which is consistent with our previous studies of stress-induced codon-biased translation in yeast [77,78].

### 3.4. The Absence of t^6^A Does Not Specifically Affect the Translation of Prion Proteins

**Figure 7:**
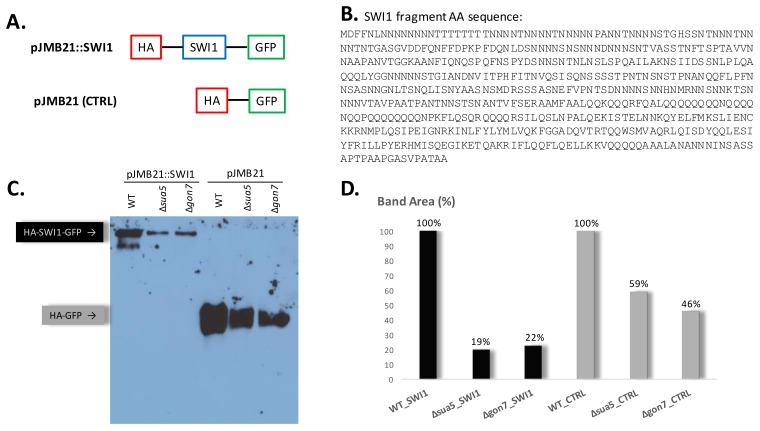
Detection of SWI1 fusion in t^6^A deficient strains. (**A**) Representation of plasmid constructs for protein expression in *S. cerevisiae*. pJMB21::SWI1 has a SWI1 fragment flanked by HA and GFP tags (N- and C-terminal, respectively). pJMB21 is used as a control plasmid and expresses the HA tag directly fused to the GFP tag. (**B**) Amino acid sequence of the expressed SWI1 fragment enriched in stretches of the t^6^A dependent codons Asn (N) and Thr (T). (**C**) Western blot detection of both fusions with an anti-HA tag antibody in WT, *sua5* and *gon7* strains. This is a representative gel of three different experiments performed with independent transformants. (**D**) Image J calculation of band intensities from a 3 h exposed film. The band intensities were converted to percentage considering, for each fusion, the WT strain intensity as 100%.

In *S. cerevisiae*, the protein with the longest stretch of codons decoded by t^6^A containing tRNAs is SWI1 with its stretch of 31 Asn and Thr amino acids starting at position 7 of the protein (24]. Because these repeats (90 nts) are longer than the RPFs (28 nts) sequenced, this gene could not be analyzed in the ribosome profiling that compared WT and t^6^A^-^ strains [36]. We set out to study if the absence of t^6^A affects the translation of proteins with long stretches of codons decoded by t^6^A-dependent tRNAs by constructing the plasmid pJMB21, which allowed for the expression of proteins containing an N-terminal HA-tag with a C-terminal GFP fusion. The N-terminal end of SWI1 (residues 1–556) was then inserted between the HA and GFP tag (Figure 7A,B). By Western blot, we compared the expression of the HA-SWI1-GFP fusion from pJMB21::SWI1 in the t^6^A single mutants *gon7* and *sua5* to the wild-type (WT) strain. And the HA-GFP fusion, expressed from pJMB21, was used as a control. In this experiment, we introduced the *gon7* mutant, whose deleted gene is part of the KEOPS complex and participates in the last step of t^6^A formation. This strain is not as crippled as the *sua5* strain and allowed us to compare two different t^6^A mutants. The expression of both fusions was detected with an anti-HA tag antibody in all three strains at the expected molecular weight (HA-SWI1-GFP with 95 kDa and HA-GFP with 33 kDa), meaning that the fusions are expressed in the WT and in the t^6^A mutants with no truncation. The intensity of the bands expressed from the t^6^A mutant strains was visually lower than the band from the WT strain, usually not detected within 1 h of membrane exposure (Figure 7C). We used Image J [83] to calculate the intensity of the bands from a 3 h exposed film. The area of the band was measured in the film and normalized based on the intensity of the respective lane in a Coomassie-stained gel run in parallel and converted to percentage considering the band intensity from the WT strain as 100% (Figure 7D). There is a smaller amount of both fusions in the t^6^A mutants compared to the WT, consisting of an approximately 80% reduction of the HA-SWI1-GFP fusion and 40–55% reduction of the HA-GFP fusion. While the gels for [^35^S]-labeling studies in Figure S3 appear to show similar protein levels in the three strains, visual interpretation of the gels is not accurate enough to distinguish modest changes in global translation. The more quantitative Western blots in Figure 7 show evidence that the absence of t^6^A modification reduces global translation by ~2-fold, at least for one representative non-codon-biased protein. This is consistent with our published ribosome profiling studies [39]. However, loss of t^6^A causes a larger reduction in the translation of proteins enriched in t^6^A dependent codons such as this SWI1 fragment.

## 4. Discussion

The ASL modifications (c)t^6^A and mcm^5^(s^2^)U, found in yeast tRNAs respectively at positions 37 and 34, are critical for correct pre-structuring of the ASL [13,14,15,16,17]. Severe diseases in humans [18,19,20,21,22] and very similar phenotypes in yeast *S. cerevisiae* [3,29,37] are observed as a result of deficiencies in both these modifications. The *sua5 elp3* mutant (lacking both t^6^A and mcm^5^U modifications), is viable in the YPD medium but cannot grow in the presence of various exogenous stressors such as elevated temperature, diamide or alternative carbon sources. Additionally, combined *sua5* and *elp3* mutations are synthetically negative, with the *sua5 elp3* double mutant presenting slower growth, morphological defects and mis-segregated nuclei compared to the *elp3* and *sua5* single mutants, resembling observations in other strains lacking critical anticodon loop modification simultaneously [29,84]. The absence of ASL modifications frequently affects translation speed and accuracy [4,39] leading to +1 frameshifting and misreading at specific codons [24,43,72]. Here we showed that t^6^A and mcm^5^U modifications contribute independently to reading frame maintenance and that their absence has an additive effect on ribosomal accuracy.

Previously, the absence of mcm^5^s2U or t^6^A in yeast was shown to induce the formation of protein aggregates [4,39]. In the present study, we detected a drastic increase in the amount of protein aggregates in the double mutant by two different methods. The presence of aggregates in morphologically aberrant cells, encompassing two or more of the non-separated individual cells may indicate that aggregate formation and extension from the mother into the daughter cell may contribute to the repeated failures in cytokinesis. Hence, synthetic growth defects in *sua5 elp3* and aberrant morphology occur along with increased protein aggregation and indications for cumulative A-site binding defects, pointing to the interdependency of these events.

Cells defective in Elongator, tRNA thiolation, and t^6^A modification commonly upregulate gene expression of Gcn4 dependent amino acid biosynthesis [3,9,39]. Gupta et al. [85] have shown that, for the tRNA thiolation defective mutant, the Gcn4 activation occurs despite the presence of elevated levels of amino acids, including Met and Cys. If this activation also occurs in *sua5* and *sua5 elp3* strains, the reduced incorporation of [^35^S] in proteins from these mutants could be a result of elevated levels of endogenous Met and Cys, which might account for reduced label incorporation via exogenous radiolabeled Met and Cys. Therefore, these results do not allow us to draw conclusions about tRNA modification-dependent protein aggregation and translation.

The quantitative proteomics of the soluble and insoluble fractions of the t^6^A mutant (*sua5* strain) shed light on the altered biological processes resulting from t^6^A absence. Among the up-regulated proteins in the mutant soluble fraction, there is an enrichment of proteins related to oxidative stress response, protein quality processing/trafficking, energy homeostasis, and mitochondrial assembly/function. The effect of t^6^A modification has been shown in human mitochondrial tRNAs (mt-tRNAs) [86] where the lack of OSGEPL1, the human homolog of yeast Qri7 involved in t^6^A formation in mt-tRNAs, resulted in reduced mitochondrial protein synthesis, impaired assembly of Complex I and respiratory defects. These effects could be a result of a dysfunction in the mitochondrial translation of the proteins ND2 and ND5, components of Complex I that contain higher frequencies of codons decoded by the five mt-tRNAs bearing t^6^A modification.

A more detailed analysis of the insoluble proteome of the t^6^A mutant (*sua5* strain) revealed a surprisingly low number of proteins with increased or decreased abundance in aggregates in the mutant compared to the WT strain, considering the expected “aggregation-prone” strain. But a closer look at the identity of the proteins enriched in the insoluble fraction revealed potentially interesting findings that will require follow-up studies. Among the proteins with increased abundance in the mutant aggregates, some are related to transcription regulation (histones H2A.2 and H2B.1, and negative cofactor 2 transcription regulator complex subunit NCB2), mitochondrial function (ubiquinol-cytochrome-c reductase subunit 7 QCR7) and oxidative stress resistance (thioredoxin peroxidase AHP1). Levels of *[PIN+]* prion protein RNQ1 were slightly increased in the aggregate pool in the mutant (1.4-fold increase, *p* = 0.07). These results suggest that the absence of t^6^A modification could affect the translation of prion proteins, as observed for SWI1.

Containing an N-terminal stretch of 31 Asn and Thr amino acids, the prion protein SWI1 is the protein with the longest stretch of codons decoded by t^6^A containing tRNAs in *S. cerevisiae* [24,51]. The detection of a fusion containing a SWI1 fragment fused to HA and GFP tags, revealed that even a protein enriched in such high number of t^6^A dependent codon stretches is fully translated in the absence of t^6^A (*sua5* and *gon7* strains), although there is a marked reduction in protein expression levels. The comparison of the HA-SWI1-GFP fusion expression to its control, HA-GFP, showed that t^6^A absence has a global effect on expression (40–55% reduction of HA-GFP compared to the WT) and an even more drastic effect on the expression of proteins enriched in t^6^A dependent codons (80% reduction of HA-SWI1-GFP compared to the WT). This marked reduction in the overexpressed SWI1 fragment contrasted with the non-significant alteration in levels of the endogenous expression of SWI1, showing in the proteomics analysis an 8% decrease in the soluble fraction and a 2% decrease in the insoluble fraction of the *sua5* strain (Table S2). Because we obtained these results using Western blot, a not a very sensitive technique, and because we did not quantify mRNA levels and hence cannot differentiate between transcription and translation effects, these findings need to be confirmed in future studies. We also need to analyze if this drastic effect in the mutant is due to reduced translation or formation of aggregates caused by misfolded proteins, or even prion formation. A reason overproduction could induce prion formation is that the increase in protein level could make it more likely for misfolding events to occur [87]. At higher local concentration it would be easier for monomers to find each other and aggregate. Prion domains (PrDs) may also be more likely to misfold when they are not in the context of the complete protein. Additionally, the increased protein levels may cause the misfolded protein to escape degradation by proteolytic pathways [87]. In the case of SWI1, its overproduction can also induce the formation of the Sup35 prion *[PSI+]* [50]. It is therefore difficult to predict whether t^6^A deficiency is detrimental or beneficial for prion formation.

## 5. Conclusions

In summary, this study showed how the critical tRNA modifications t^6^A37 and mcm^5^s^2^U34 contribute to the maintenance of proteome integrity of the model yeast *S. cerevisiae* by demonstrating the effects caused by the deficiency of both modifications in the cell. While elevated +1 frameshift levels were observed in single *sua5* and *elp3* mutants (t^6^A- and mcm^5^s^2^U-deficient, respectively) showing that each modification independently contributes to the reading frame maintenance, a further increase in +1 frameshift levels in the *sua5 elp3* double mutant demonstrated an additive translation defect. Synthetic phenotypes in the double mutant such as slower sporulation, enhanced growth defect, aberrant morphology, and synthetic lethality to various exogenous stressors reinforced the additive and possibly synergistic translation defects. The combined translation defects resulting from the lack of both modifications reflect also in the formation of protein aggregates in the cell. Although aggregates have been previously detected in the single mutants, a strong increase was observed in the double mutant, particularly in morphologically aberrant cells. Proteomics of the *sua5* single mutant indeed revealed an increase in the abundance of proteins associated with protein folding and trafficking, as well as with mitochondrial function, oxidative stress response, and energy homeostasis in t^6^A-deficient strain. These results point to a global mistranslation effect leading to protein aggregation, although without regard to t^6^A-dependent or biased codon usage in parent genes. This work reinforces the importance of the complex modification of the ASL in assuring the accuracy and efficiency of the translation process.

## Figures and Tables

**Table 1:** *Saccharomyces cerevisiae* strains used in this study.

Strain	Genotype	References/Sources
BY4741	*MAT*a, *his3Δ, leu2Δ, met15Δ, ura3Δ*	Euroscarf, Frankfurt
BY4742	*MATα*, *his3Δ, leu2Δ, lys2Δ, ura3Δ*	Euroscarf, Frankfurt
Y02742	BY4741 *elp3::KANMX4*	Euroscarf, Frankfurt
RK311	BY4741 *HSP104-GFP::natMX6*	This study
RK477	BY4741 *elp3::SpHIS5*	This study
VDC9100	BY4742 *sua5::KANMX4*	[59]
Y07017	BY4741 *gon7::KANMX4*	Euroscarf, Frankfurt [39]
RK340	BY4742 *sua5::KANMX4 elp3::SpHIS5*	This study
RK357	BY4742 *sua5::KANMX4 elp3::SpHIS5 HSP104-GFP::natMX6*	This study
RK359	BY4742 *elp3::SpHIS5 HSP104-GFP::natMX6*	This study
RK360	BY4742 *sua5::KANMX5 HSP104-GFP::natMX6*	This study
LPO0180	BY4741 pJMB21	This study
LPO0181	BY4741 pJMB21::SWI1	This study
LPO0085	BY4742 *sua5::KANMX5* pJMB21	This study
LPO0087	BY4742 *sua5::KANMX5* pJMB21::SWI1	This study
LPO0089	BY4741 *gon7::KANMX5* pJMB21	This study
LPO0091	BY4741 *gon7::KANMX5* pJMB21::SWI1	This study

**Table 2:** Gene ontology (GO) category enrichment for soluble proteins that are overrepresented in *sua5* relative to WT (Fold-change *sua5*/WT > 1.3) - *Function.*

Go Category	# Genes	*P*-Value
Protein binding involved in protein folding	7	6.53 × 10^−8^
Misfolded protein binding	7	4.24 × 10^−7^
Heat shock protein binding	7	3.37 × 10^−6^
ATPase activity, coupled	12	6.40 × 10^−4^
Purine ribonucleotide triphosphate binding	25	1.23 × 10^−3^
Unfolded protein binding	8	2.08 × 10^−3^

**Table 3:** Summary of soluble proteins that are significantly (*p* < 0.05) overrepresented in *sua5* relative to WT and map to enriched GO categories.

Protein	ORF	Description	Fold-Change
Mitochondrial heat shock protein, SSC3	YEL030W	Refolding imported precursors	3.0
rRNA methyltransferase 2, mitochondrial	YGL136C	Peptidyl transferase domain	2.3
Exosome complex component RRP40	YOL142W	Exoribonuclease	1.9
Mitochondrial heat-shock protein SSC1	YJR045C	Binds to precursor preprotein	1.8
Interacting with cytoskeleton protein 1 ICY1	YMR195W	Required for the viability of cells lacking mtDNA	1.7
Plasma membrane ATPase 2	YPLO36W	Nutrient active transport by H+ symport	1.7
rRNA-processing protein CGR1	YGL029W	Involved in nucleolar integrity, required for processing 60S pre-RNA	1.7
Mitochondrial import receptor subunit TOM5	YPR133W-A	Component of receptor complex responsible for recognizing, translocating cytosolically synthesized mitochondrial preproteins	1.7
Endoplasmic reticulum chaperone BiP (aka KAR2)	YJL034W	Role in facilitating assembly of multimeric protein complexes in ER—required for secretory polypeptide translocation	1.7
Cytochrome b-c1 complex subunit 10 QCR10	YHR001W-A	Part of the mitochondrial respiratory chain that generates electrochemical potential coupled to ATP synthesis	1.7
V-Type proton ATPase subunit B	YBR127C	Non-catalytic subunit of V-ATPase: electrogenic proton pump generating proton motive force of 180 mV	1.7
Sulfiredoxin	YKL086W	Contributes to oxidative stress resistance by reducing cysteine-sulfinic acid formed by oxidants in the peroxiredoxin TSA1	1.6
Vacuolar morphogenesis protein 10	YOR068C	Required for vacuolar fusion; involved in the early steps of the fusion pathway	1.6
Threonine-tRNA ligase, mitochondrial	YKL194C	-	1.6
Mitochondrial peroxiredoxin PRX1	YBL064C	Involved in mitochondrial protection from oxidative stress	1.6
Inheritance of peroxisomes protein 1	YMR204C	Inhibition of peroxisomes	1.6
Elongation factor 3A	YLR249W	Release of deacylated tRNA from ribosomal E-site during synthesis	1.6
Heat shock protein SSA2	YLLO24C	Transport polypeptides both across the mitochondrial membranes and into the ER	1.6
Glutathione peroxidase-like peroxiredoxin 2 GPX2	YBR224W	Protects cells from phospholipid hydroperoxides and nonphospholipid peroxides during oxidative stress	1.6
Glutathione peroxidase-like peroxiredoxin HYR1	YIRO37W	Oxidative stress response pathway	1.4
ATP synthase subunit f, mitochondrial	YDR377W	Mitochondrial membrane ATP synthase	1.4
Heat shock protein SSA1	YALOO5C	Role in the transport of polypeptides both across the mitochondrial membranes and into the endoplasmic reticulum	1.4

**Table 4:** STRING functional enrichment analysis of genes that only use AGA or AGG codons for arginine. The observed gene count is the number of genes from the target list found in each functional category, with the background gene count describing the total number of genes found in the category.

GO Term ID	Term Description	Observed Gene Count	Background Gene Count	False Discovery Rate
GO:0046034	ATP metabolic process	31	94	2.55 × 10^−12^
GO:0009167	purine ribonucleoside monophosphate metabolic process	32	118	1.74 × 10^−11^
GO:0009161	ribonucleoside monophosphate metabolic process	33	136	6.23 × 10^−11^
GO:0022900	electron transport chain	25	74	1.09 × 10^−10^
GO:0009150	purine ribonucleotide metabolic process	32	147	1.29 × 10^−09^
GO:0006119	oxidative phosphorylation	18	39	2.27 × 10^−09^
GO:0009259	ribonucleotide metabolic process	33	162	2.27 × 10^−09^
GO:0019693	ribose phosphate metabolic process	35	182	2.27 × 10^−09^
GO:0042775	mitochondrial ATP synthesis coupled electron transport	17	37	7.14 × 10^−09^
GO:0022904	respiratory electron transport chain	18	45	1 1.07 × 10^−08^
GO:0009117	nucleotide metabolic process	39	250	2.69 × 10^−08^
GO:1902600	proton transmembrane transport	25	108	4.38 × 10^−08^

## Supplementary Materials

The following are available online at https://www.mdpi.com/2218-273X/10/2/322/s1, Figure S1: Map of pJMB21; Figure S2; Hsp104-GFP signals in *sua5* and *elp3* mutant backgrounds carrying a genomic HSP104-GFP fusion. Figure S3: Quantification of aggregation in wild-type (WT), *sua5*, and *sua5 elp3* strains by labeling proteins with [^35^S]-methionine and [^35^S]-cysteine; Figure S4; Gene Ontology enrichment analysis of proteomics datasets; Figure S5: Quantification of the physicochemical properties of significantly up- and down-regulated proteins in the soluble and insoluble fractions from WT and *sua5* strains.; Figure S6: AGN-AGN dicodons analysis, Table S1: Oligonucleotides used. Table S2: Proteomics data for wild-type and *sua5* mutant strains. See References [69,76].
